# Supplementary material for: Barriers and facilitators to satisfaction with diabetes care: The perspectives of patients attending public diabetic clinics in Dar es Salaam, Tanzania
Source: PLoS One. 2024 May 9;19(5):e0302858. doi: 10.1371/journal.pone.0302858 (PMC11081265; doi:10.1371/journal.pone.0302858)
Supplement: S1 Text — (DOCX) [file pone.0302858.s001.docx]

**A guide of in-depth interview for patients’ perceived barriers and facilitators towards satisfaction with diabetes care at the clinics**

1. What aspect of care do you receive when you come to your diabetic clinic? (Probe type of treatments and other services given to the patient)
2. How do health workers interact with you during the time you are seeking care at this clinic? (Probe the aspects of communication and technical expertise and why he or she thinks that way)
3. What do you think about the availability or accessibility of the services at this clinic by the time you need the treatments? (Probe the time travel to access the facility and costs of services provided)
4. What do you think about the adequacy of care you receive from the health facility (diabetes clinic) as far as your diabetic status is concerned? (Probe the days the clinics operates and are they enough?, also if he/she gets all the services desired or expected)
5. What do you think could be the barriers that limit the provision of quality care at this clinic? (Probe personal and facility-related challenges and how they affect the care delivery)
6. What do you think facilitates/helps the provision of quality care at this clinic? (Probe personal and facility-related facilitators/enablers and how they affect the care delivery)
7. Is there anything more you would like to add?

**Thank you for the cooperation!**
