# Supplementary material for: Barriers and facilitators to satisfaction with diabetes care: The perspectives of patients attending public diabetic clinics in Dar es Salaam, Tanzania
Source: PLoS One. 2024 May 9;19(5):e0302858. doi: 10.1371/journal.pone.0302858 (PMC11081265; doi:10.1371/journal.pone.0302858)
Supplement: S2 Text — (DOCX) [file pone.0302858.s002.docx]

**Barriers and facilitators to satisfaction with diabetes care: the perspectives of patients attending public diabetic clinics in Dar es Salaam, Tanzania**

| **Table 1.** Socio-demographic characteristics of study participants | |
| --- | --- |
| **Variables** | **Frequencies, n(%)** |
| **Age of participant** |  |
| 26-45 years | 6(17.2) |
| 46-65 years | 20(57.1) |
| 66-85 years | 9(25.7) |
| **Sex of participant** |  |
| Male | 19(54.3) |
| Female | 16(45.7) |
| **Duration of diabetes since diagnosis** |  |
| 1-10 years | 22(62.9) |
| 11-20 years | 11(31.4) |
| 21-30 years | 2(5.7) |
| **Participant’s marital status** |  |
| Single | 6(17.2) |
| Married | 11(31.4) |
| Widow/widower | 11(31.4) |
| Divorced | 7(20.0) |
| **Participant’s level of education** |  |
| No formal education | 5(14.3) |
| Primary education | 16(45.7) |
| Secondary education | 9(25.7) |
| College/higher education | 5(14.3) |
| **Participant’s occupation** |  |
| Formally employed | 6(17.2) |
| Self-employed | 8(22.9) |
| Unemployed | 16(45.7) |
| Retired | 5(14.3) |
| **Time since enrollment at the clinic** |  |
| < 6 months | 5(14.3) |
| 6-12 months | 12(34.3) |
| > 12 months | 18(51.4) |
| **Average travel time from home to health facility by car** |  |
| < 1 hour | 10(28.6) |
| 1-3 hours | 17(48.5) |
| > 3 hours | 8(22.9) |
| **Average duration of time spent at the facility per visit** |  |
| < 1 hour | 6(17.2) |
| 1-3 hours | 12(34.3) |
| > 3 hours | 17(48.5) |

**Excerpts of transcripts**

**Financial constraints**

“As we are speaking today, I have sold my little house I had there in Kiluvya because I was heavily indebted and most of the expenses were attributed to these medicines I’m using. I’m using three kinds of medications and all of them are very expensive for someone like me to purchase because I solely depend on relatives to fund my treatments and most of them are just peasants there in Visiga” (Participant 5).

“It has been two years now without my leg, during those years I used to cultivate maize and vegetables to get food and earn little amount of money for catering to other basic needs. Currently, as you can see my son, I can’t do all those activities, and thereof I depend on my children for everything including hospital expenditures” (Participant 29).

“With the health insurance card I had, I had to use an extra amount of money to buy some of the drugs because doctors here told me that my card couldn’t afford all of them. Also, some of these drugs are sometimes only available at street private pharmacies and are very expensive to purchase. I wish to have those NHIF cards because I’m being told that those are at least comprehensive meaning that they can buy all the drugs I’m using currently” (Participant 13).

“My relatives are a little bit upset now because I routinely ask them for money to buy the medicines I’m being prescribed with. Some of my close relatives have developed arguments to discourage others who help me some amount of money for attending the clinics by claiming that, they will run bankrupt for treating an incurable disease” (Participant 21).

“The government don’t offer any significant support to diabetic patients particularly financially to enhance us with the capability to pursue the healthcare services……. This clinic for instance is owned by the government but can you imagine we have to pay consultation fees each day we come here to get the services” (Participant 32).

“In my point of view, the government has neglected us unlike those people with HIV/AIDS as you know that those people are being offered free medicines to take daily and are sometimes empowered with balanced diet at their treatment centres” (Participant 18).

“The strips I’m getting on each visit are not enough for monitoring the blood sugar until the next clinic visit. So, there are days and situations when we inject insulin without measuring the blood sugar levels……. they always tell us that the strips are very few for each to get the required actual number monthly considering the number of patients in need, So, they have to distribute few strips so that everyone can get” (Participant 15).

“It is very difficult sometimes to have the insulin injection regularly because I think you know what can happen with it when you inject blindly (without measuring the current blood sugar). All these are attributed to lack of enough strips for measuring the blood sugar” (Participant 20).

“I don’t know if there is no outstanding supply of these medicines to these hospital pharmacies or if there are other issues behind because many times, I’m being directed to the external pharmacy to access some of the medicines. And keep in mind that in those outside private pharmacies, the prices for these medicines are higher than those of in-hospital pharmacies” (Participant 23).

**Unfavorable clinic environments**

“The waiting area here as you can see is not friendly at all, firstly, the number of benches is not sufficient to accommodate the actual population of patients waiting for services here. Secondly, this place is poorly ventilated to the extent that we have to sometimes get out there to ventilate especially when the queue is very long” (Participant 11).

“I advise that they have to modify this waiting area by adding more benches for patients to sit down and putting air conditioners around. The latter will at least enable us to wait for a long time before consultations tireless……... I have said because I actually know it is not easy to employ more healthcare providers urgently considering our country’s economic constraints but modifying the waiting area first is very possible” (Participant 16).

“We acknowledge the presence of computerized care delivery systems, yes but the system sometimes is very boring because you can wait for 1-2 hours the network is not working and we have to wait for it without getting treatments” (Participant 1).

“The issues of internet problems here are very common, they are very repetitive to the extent that we have to spend more time to get the treatments. Therefore, it interferes with other work routines because we are being late here despite coming earlier in the morning” (Participant 35).

“Today, one of my friends has recently left untreated because of network problems. He promised to return tomorrow for the services because with such a network problem and high patient load, as you can see, he would attend very late to his works there in the market” (Participant 28).

“As you have said in any success, barriers are obvious. In my point of view concerning this aspect, the barriers are like a shortage of care providers which makes us delay getting the services. I wish we could have more specialized doctors and consultation rooms, it could be better than this situation” (Participant 33).

“Eeeeeh listen, we diabetic patients sometimes suffer from the long waiting time especially when the blood glucose levels are elevated or lowered below normal ranges, we feel very uncomfortable and you find such a situation on your clinic appointment day, can you imagine how you are going to suffer the consequences……... During my clinic visit here in January, I fainted here while waiting for my queue to see the doctor and therefore, I was admitted to the ward that day I didn’t go back home as I used to do” (Participant 9).

**Good provider-patient relationships**

“In this clinic, we are usually empowered and encouraged to give out our views on the treatments we are getting during the consultations. The doctors here give us that ability for making decisions regarding our health regardless of the situation because they usually tell us that health services are delivered only upon consent from patients” (Participant 4).

“Yes, as you have said there are had situations we encounter here. For instance, during my last visit here I was unable to cover all the expenses plus the new medications my doctor prescribed for the first time. I rejected them because I had no money to purchase them but the doctor emphasized their importance because my blood sugar was very high. Social workers were then summoned by the doctor and after a couple of discussions, I was offered the medications with promise to compensate the expense on my subsequent visits” (Participant 21).

“By the day I moved from my local clinic at the dispensary and came here to Temeke, I felt a big difference between the two contexts. At our dispensary, we attended the clinic just to get prescriptions to buy the medicines…Aaaah I mean there was no room for getting time to sit with nurses and doctors and explaining to them what has been going on with you from the last visit, unlike here we get time to sit together with them and they support us so that we are motivated to continue with treatments” (Participant 31).

“With the aspect of support from the experts here, I can guarantee you that there is good support from the nurses and doctors not only through giving us these medicines like what I have here but also, they get time to talk with us and explore us a lot……. Psychologically? Yes, there are days we come here with unusually elevated blood sugar to the extent that we are disappointed with taking the medications but the nurses and doctors do encourage us to continue with them by telling us that it’s sometimes normal to have such ups and downs in the maintenance of blood sugar” (Participant 7).

“In the doctor’s room, they usually let us explain our concerns and after those clarifications are made, they give us feedback according to their knowledge and experience because you know what? What they tell us is what they have studied for many years in the class and they faced with other patients in their daily routines here at the clinic. Therefore, in my point of view there is good communication between them and us patients like what I have said they listen to us carefully and respond to our questions and we are satisfied with that” (Participant 30).

“It was yesterday in the mid-day, I experienced a persistent headache after taking Metformin drugs, I think you know them……... after telling my daughter about the situation, she advised me to phone Dr …….. because I had the numbers in my mobile phone and my daughter also had them. So, I spoke with him through the phone and he urged me to attend the clinic today despite that it was not my clinic day” (Participant 2).

“Yes, there is this number we have been encouraged to call it in case we have anything we don’t understand. It is a customer care mobile phone number for helping patients when they are far away from these hospital premises. Despite that it operates with normal call charges, it helps many of us and we are happy with the response they give us through the phone” (Participant 14).

**Continuity of care**

“The system nowadays is very organized despite some minor challenges, the information is fed and saved in the computers for future use. For example, with the system they use here, they can track back the trend of your blood sugar upon each visit and see how it goes because it has been stored there. The prescriptions are also uploaded online so that the pharmacists can access them and dispense the medications easily” (Participant 9).

“In the past years, the system was very poor because we had a lot of unnecessary movements from one room to another……... I mean there was more paperwork and we used to trace the availability of medicines physically unlike today. For example, there were situations, when the doctor prescribed medications and directed you to go to the pharmacy to get the drugs, but once you reached the pharmacy you are being told that such drugs were not there, you see? So, you have to go back to the doctor and tell him/her that the prescribed drug is not there………Nowadays, the doctor can visualize the available drugs through the network system they use and if the drug is not there, he/she changes it or he/she directs you where you can access the required drugs” (Participant 3).

“……. Yes, we are being counselled here on diet and meal plans so that we can sustain the balance of blood sugar. As you can see there on that board, they have printed those learning materials which are easier for us to understand how things are supposed to be and we are being counselled each visit here on how we can maintain our health through dietary modification and compliance with medications……the plans do vary of course depending on individual condition because each one has his/her unique challenges with this disease” (Participant 24).

“Every day here at 6 a.m., teaching sessions are being conducted by nurses and the topics are not the same each day. They usually establish and share a timetable for what will be taught on a particular day. For example, today we had a session on diabetic foot syndromes, so you can see we have been encouraged not to wear tight shoes to avoid such complications” (Participant 17).

“We usually set goals collaboratively for maintenance of blood sugar, the healthcare providers do emphasize adherence to medications and dietary counselling sessions are conducted so that we can have a meal plan for regulating the blood sugar levels……... the plans are usually monthly based because evaluation is done monthly upon each clinic attendance” (Participant 34).

“I can say nowadays things have changed a lot, in those years when we were attending the clinic there in Upanga, we had no freedom like what we are having in these recent few years. I can tell you, in those years during consultations, doctors and nurses provided healthcare services in this clinic as orders to implement. We had no chances to opt for alternative drugs when something was wrong with the medications we had” (Participant 10).
