# Supplementary material for: Barriers and facilitators to satisfaction with diabetes care: The perspectives of patients attending public diabetic clinics in Dar es Salaam, Tanzania
Source: PLoS One. 2024 May 9;19(5):e0302858. doi: 10.1371/journal.pone.0302858 (PMC11081265; doi:10.1371/journal.pone.0302858)
Supplement: Attachment — Submitted filename: Response to Reviewers.pdf [file pone.0302858.s004.pdf]

April 02, 2024

To

Editor-in-Chief

PLOS ONE Journal

Dear Sir/Madam,

On behalf of my co-authors, I am grateful for the valuable and constructive review of our manuscript **PONE-D-24-00042R1** entitled **“Barriers and facilitators to satisfaction with diabetes care: the perspectives of patients attending public diabetic clinics in Dar es Salaam, Tanzania”**. We have revised the manuscript according to reviewer’s comments. The specific response to reviewer’s comments is elaborated in details within the matrix response sheet below after this letter.

Yours Sincerely,

E.Chona

....

Emmanuel Z. Chona (*Corresponding Author)*

E-mail: emmanuelchona20@gmail.com

Matrix response sheet on academic editor and reviewers' comments to manuscript **PONE-D-24-00042R1** entitled **"*Barriers and facilitators to satisfaction with diabetes care: the perspectives of patients attending public diabetic clinics in Dar es Salaam, Tanzania*"**

**Reviewer's comments**	**Response from author**
Please make sure to replace "diabetic patients" with "people with diabetes". Some statements have not been changed eg lines 33 and 563	We have revised the manuscript and replaced the phrases "diabetic patients" with "people with diabetes".<br>Please see the revised manuscript (page 2 line 32, page 30 line 563, and page 34 line 633).
Page 7 line 120 – The study was a qualitative descriptive study and not a phenomenological one. Please remove "phenomenological approach"	The phrase "phenomenological approach" has been removed from the revised manuscript.<br>Please see the revised manuscript (page 7 line 120-122).
